# Infectious Disease Surveillance in the Era of Big Data and AI: Opportunities and Pitfalls

**DOI:** 10.7759/cureus.93929

**Published:** 2025-10-06

**Authors:** Courage O Idahor, Ena-Jane O Esomu, Ndidiamaka Ogbonna, Zaafirah Momoh, Omo A Ogbeide, Osamagbe Ikhu-Omoregbe, Augustina Adigwe, Osayuwamen M Erhabor, Osamagbe Osaghae, Nosa Orons

**Affiliations:** 1 Emergency Medicine, Nottingham University Hospitals NHS Trust, Nottingham, GBR; 2 Surgery, University of Benin, Benin City, NGA; 3 Family Medicine, Edo State University Uzairue, Uzairue, NGA; 4 Obstetrics and Gynecology, Asaba Specialist Hospital, Asaba, NGA; 5 General Practice, NES Healthcare UK, Aylesbury, GBR; 6 Internal Medicine, University of Benin, Benin City, NGA; 7 General Medicine, Zaporozhye State Medical University, Zaporozhye, UKR; 8 Emergency Medicine, University of Benin, Benin City, NGA

**Keywords:** artificial intelligence in medicine, big data, geospatial data, infectious disease surveillance, machine learning (ml)

## Abstract

The landscape of infectious disease surveillance (IDS) is undergoing a profound shift, driven by the rapid emergence of big data and artificial intelligence (AI). Traditional surveillance systems, while foundational to public health, are increasingly limited by delayed reporting, data silos, and fragmented information flows. In response to these limitations, the integration of AI and big data offers new possibilities for enhancing disease detection, monitoring, and response strategies on both local and global scales.

This review explores the potential of AI-enabled tools and big data systems to support early outbreak detection, real-time surveillance, and predictive modeling. These technologies facilitate the synthesis of diverse datasets, including clinical, genomic, geospatial, and environmental information, enabling a more holistic understanding of disease patterns. Additionally, AI contributes to improved diagnostic accuracy and optimized resource allocation, which are critical during public health emergencies.

However, the adoption of these technologies has not been without challenges. Concerns about data privacy, equity in access, algorithmic bias, and over-reliance on automated systems present significant ethical and operational hurdles. In low-resource settings, limited digital infrastructure further complicates implementation. The review also highlights real-world applications from recent outbreaks, such as COVID-19, influenza, and Zika, to demonstrate both the promise and the limitations of AI-driven surveillance.

To move forward responsibly, public health systems must adopt a balanced approach that integrates AI capabilities with human oversight. Strategic investment, cross-sector collaboration, and the development of clear ethical frameworks are essential to unlocking the full potential of big data and AI in infectious disease surveillance.

## Introduction and background

Infectious disease surveillance (IDS) serves as a key tool in epidemiology, encompassing the systematic collection, analysis, and interpretation of extensive data to detect disease trends, recognize outbreaks, identify emerging pathogens, and support efforts in disease monitoring and eradication [1,2]. The World Health Organization (WHO) advocates for the concept of integrated disease surveillance as a core strategy to strengthen national disease monitoring systems [3,4], a concept that was later renamed Integrated Disease Surveillance and Response (IDSR) [4].

Traditional and disease-specific surveillance relies on information reported by healthcare facilities and diagnostic laboratories. These systems collect structured, predefined information about infectious disease events. However, this indicator-based surveillance (IBS) is often inefficient due to constraints in resources, time, and reporting systems. As a result, it frequently produces incomplete data on emerging infectious diseases [5-7].

In response to these limitations, emerging technologies such as advanced analytics, artificial intelligence (AI), the Internet of Things (IoT), remote sensing, and molecular tools offer the potential to enhance surveillance capabilities. These innovations facilitate real-time monitoring, early anomaly detection, and the improved prediction of outbreak risks [8-11]. Moreover, tools such as point-of-care (POC) diagnostics, telemedicine, and digital contact tracing can significantly increase the timeliness and efficiency of outbreak response efforts [11-13].

AI and big data have become transformative tools in the realm of infectious disease surveillance. These technologies enable the collection and analysis of information related to transmission patterns, risk factors, and clinical outcomes. By leveraging these capabilities, healthcare professionals can identify high-risk populations, trace the spread of diseases, and predict potential outbreaks. This enhances early intervention strategies and optimizes resource allocation [14,15].

The utility of AI and machine learning (ML) has been particularly evident during the COVID-19 pandemic. These tools have been widely adopted for tasks such as vaccination tracking, contact tracing, and the implementation of nonpharmaceutical interventions such as movement restrictions. In China, for instance, health QR codes were integrated into popular mobile applications such as Alipay and WeChat. These enabled real-time evaluations of transmission risk in public areas and offered access to AI-powered medical chatbots for health-related queries [16,17].

The integration of big data further strengthens surveillance systems and contributes to improved pandemic preparedness. When real-time digital data from sources such as health applications, electronic medical records, social media, search engines, and mobile networks are combined with spatial and temporal information, public health assessments can be significantly improved. Numerous studies highlight the effectiveness of such real-time data in monitoring health trends [18-24]. Several of these studies have been specifically applied to monitor and predict epidemics such as COVID-19, Zika, Ebola, and influenza [18-20,24,25].

Amid rising global health threats, particularly those posed by emerging and re-emerging infectious diseases, the WHO continues to play a crucial role in coordinating international preparedness and response. A core element of this mandate involves strengthening health emergency preparedness, response, and resilience (HEPR) through effective global governance. This strategy includes promoting leadership, inclusivity, and accountability among member states, underpinned by legal instruments such as the International Health Regulations (2005) [26,27].

In alignment with these efforts, the Global Health Security Index (GHSI) was developed to evaluate the preparedness and capacity of 195 countries in responding to health threats [28,29]. Jointly created by the Nuclear Threat Initiative, Johns Hopkins Center for Health Security, and Economist Impact, the GHSI functions as a comprehensive benchmarking tool [29-31]. First published in 2017, the index assesses national health security across six core categories, incorporating 37 indicators and 171 questions [29,30]. Beyond technical capacity, the GHSI also considers political context and compliance with international health regulations, offering a multidimensional perspective on health system resilience [29,32]. By guiding governments in strategic planning and resource allocation and by encouraging periodic reassessment every two to three years, the GHSI aims to strengthen global preparedness, support policy reform, and reduce the risk of future health emergencies [29,33].

Given the rapid evolution of technology and its implications for global health threats, scholars have explored hybrid models that integrate tools such as blockchain, IoT, big data, and AI [34,35]. These innovative models aim to enhance disease prevention and monitoring through proactive, technology-driven strategies, and they hold transformative potential for managing outbreaks such as COVID-19 [35].

This narrative review aims to examine how big data and AI are transforming infectious disease surveillance. It focuses on identifying key opportunities to improve early detection, monitoring, and response while also critically evaluating the limitations, ethical concerns, and implementation challenges associated with these technologies.

One promising opportunity in this domain is the use of AI-powered chatbots. These systems leverage natural language processing (NLP) and speech synthesis to deliver free primary healthcare education, information, and advice. Through these platforms, epidemic prevention knowledge can be widely disseminated to the public [36,37].

Despite their benefits, AI technologies also raise significant ethical and privacy concerns. Their application in public health surveillance requires the careful management of sensitive health information. The protection of data privacy and security is therefore of paramount importance [38,39].

The deployment of AI in managing infectious diseases presents various ethical and legal challenges. These include issues of health equity, social justice, patient autonomy, and data confidentiality. Because AI systems often depend on large volumes of personal health data, there are ongoing concerns about the privacy of sensitive medical information. Even when data are anonymized and shared with third-party aggregators, the risk of re-identification remains due to sophisticated data linkage techniques [15,40].

Data privacy is a particularly critical concern in the context of AI and big data in public health. Although these technologies have the potential to improve health outcomes, they also pose risks to individual rights if data are not adequately protected. Health information is inherently sensitive, and its misuse can lead to identity theft, discrimination, and the erosion of public trust. While anonymization is helpful, the potential for re-identification continues to present an ethical challenge [41,42].

## Review

Conceptual framework

Infectious disease surveillance (IDS) serves as the cornerstone of public health systems, providing the intelligence required to detect outbreaks, monitor epidemiological trends, and guide interventions. Defined broadly, IDS is the systematic collection, analysis, interpretation, and dissemination of health data essential to the planning, implementation, and evaluation of public health practice [43]. This function is critical not only for the control of known diseases but also for the timely identification of emerging pathogens. Traditional surveillance frameworks have typically relied on case reporting from healthcare facilities, sentinel site monitoring, laboratory confirmations, and national health registries [44]. While these systems have historically proven valuable, they often suffer from delays in data reporting, the underreporting of cases, fragmented information systems, and limited capacity for real-time analysis [45].

The shortcomings of conventional IDS became glaringly evident during global health emergencies such as the 2014 Ebola outbreak in West Africa and the early stages of the COVID-19 pandemic. In both cases, the delayed recognition of clusters of disease hindered timely response and contributed to significant morbidity and mortality [46]. These gaps have prompted a reimagining of surveillance strategies, leveraging technological innovations to overcome historical limitations. A central part of this shift has been the integration of big data and artificial intelligence (AI) into surveillance architectures.

Big data in public health refers to the voluminous and complex datasets generated from diverse and often nontraditional sources, including electronic health records (EHRs), mobile devices, genomic sequencing, social media, environmental sensors, and population mobility data [47]. These data streams present an unprecedented opportunity to enhance disease surveillance by capturing granular and real-time insights into health behaviors, symptomatology, and pathogen transmission dynamics. Unlike traditional datasets that are often siloed and structured, big data is characterized by its volume, velocity, variety, veracity, and value: the so-called five Vs [48]. This multidimensionality enables a richer, more dynamic understanding of public health landscapes, provided the appropriate analytical tools are employed.

Artificial intelligence offers these analytical tools. As a discipline encompassing a suite of computational techniques, AI includes machine learning (ML), deep learning, natural language processing (NLP), and predictive analytics, all of which can transform raw data into actionable intelligence. ML algorithms, for instance, can be trained to detect patterns in syndromic data or predict outbreaks based on historical and environmental variables [49]. NLP can process and extract relevant information from unstructured clinical notes or social media posts, thus broadening the surveillance net beyond conventional clinical settings [50]. Predictive analytics, which combines historical data with real-time inputs, can forecast disease spread and estimate the impact of interventions, enabling more proactive public health responses [51].

Advancements in technology have also led to the development of integrated digital platforms and mobile-based surveillance tools, particularly in low-resource settings. Programs such as the Global Public Health Intelligence Network (GPHIN) and HealthMap demonstrate the early adoption of big data approaches in global surveillance. GPHIN, launched by the Public Health Agency of Canada, uses NLP to analyze online news for early signs of disease outbreaks and was instrumental in raising initial alerts during the 2003 SARS outbreak [52]. HealthMap similarly aggregates and analyzes data from diverse online sources, including news websites, blogs, and official alerts, to provide real-time information on infectious disease events [53].

More recent innovations include AI-powered platforms such as BlueDot and Metabiota, which utilize airline ticketing data, climate patterns, and news reports to model disease transmission and assess pandemic risk. Notably, BlueDot flagged the novel coronavirus outbreak in Wuhan before the World Health Organization (WHO) officially acknowledged it [54]. These cases highlight the transformative potential of integrating big data and AI into surveillance systems, capabilities that are especially crucial in an era marked by the increasing emergence of pathogens and global interconnectedness.

The transition from traditional to AI-enhanced surveillance is not without its complexities. Traditional surveillance, typically passive in nature, depends heavily on health worker compliance and timely data entry. Active surveillance, though more robust, is resource-intensive and often constrained by geographic and logistical limitations [55]. AI-enhanced systems mitigate these challenges by automating data capture and analysis, allowing for near-real-time situational awareness. For example, automated EHR surveillance can identify anomalous symptom clusters across hospitals, flagging them for investigation before they escalate into outbreaks [56]. Similarly, the geospatial analysis of mobile phone data has been used to track population movement and predict areas at high risk of disease transmission, as demonstrated during the Zika virus epidemic in the Americas [57].

Incorporating AI into surveillance also allows for more nuanced risk stratification. For instance, ML models have been used to stratify patients with COVID-19 by their risk of hospitalization based on preexisting conditions, demographic data, and early symptoms, informing clinical and public health decision-making [58]. AI tools also facilitate real-time dashboards and alerts, improving transparency and enabling rapid decision-making during crises. The Johns Hopkins University COVID-19 dashboard, which relied on multiple big data inputs, exemplifies how timely and accessible surveillance data can inform global and local response strategies [59].

Nevertheless, the implementation of AI-driven IDS requires robust digital infrastructure, legal frameworks for data sharing, and ethical considerations concerning data privacy and algorithmic bias [60]. Data heterogeneity, interoperability issues, and limited technical capacity in many regions can hamper integration efforts [34]. Additionally, while AI can enhance pattern recognition and prediction, it cannot replace the need for expert interpretation and ground-level epidemiological investigation. Therefore, a hybrid model that synergizes traditional surveillance principles with AI capabilities is emerging as the gold standard [61].

In sum, the conceptual landscape of infectious disease surveillance is undergoing a paradigm shift catalyzed by the rise of big data and artificial intelligence. While traditional methods laid the groundwork for systematic monitoring and outbreak response, their limitations have necessitated more dynamic and integrative approaches. Big data, with its vast scale and diverse origins, coupled with AI’s analytical power, holds promise for more responsive, predictive, and inclusive surveillance systems. This transformation, however, must be pursued with attention to infrastructure, equity, data governance, and interdisciplinary collaboration to realize its full potential.

Opportunities: The potential of big data and AI in infectious disease surveillance

The integration of big data and artificial intelligence (AI) into infectious disease surveillance systems presents a transformative opportunity to revolutionize public health responses through early detection, predictive modeling, real-time monitoring, and resource optimization. These technologies have shifted the paradigm from reactive to proactive public health surveillance, equipping systems with the capacity to anticipate outbreaks, track disease dynamics in real time, and guide precise interventions based on a multitude of data streams. Their growing role in global health security underscores the potential for more responsive, scalable, and effective disease control strategies.

One of the most significant contributions of big data and AI to infectious disease surveillance lies in their capacity for enhanced early detection and predictive modeling. Machine learning (ML) algorithms and AI-driven models can detect subtle anomalies in large datasets that might signal the beginning of an outbreak. For example, ML models trained on electronic health records (EHRs), syndromic surveillance inputs, and historical outbreak patterns have demonstrated the ability to forecast influenza activity several weeks in advance, outperforming traditional surveillance systems in accuracy and timeliness [62]. Such predictive capabilities extend beyond seasonal flu, with similar models being applied to detect and track emerging threats such as dengue, Zika, and COVID-19 [63]. AI systems such as BlueDot and HealthMap use natural language processing (NLP) to analyze online data sources, such as news reports and airline ticketing information, to identify clusters of unusual disease activity, sometimes before official agencies report them [64].

Predictive analytics, a subfield of AI, enables the forecasting of disease trajectories and the identification of high-risk zones based on historical and environmental data. This modeling was especially valuable during the COVID-19 pandemic, where predictive models were helpful in estimating hospital burden, planning lockdown measures, and allocating healthcare resources based on predicted case surges [65]. By integrating geospatial data, climate variables, and human mobility patterns, these tools provide granular insights into how diseases may spread within and across borders. This capability allows health authorities to initiate targeted interventions in specific regions, potentially mitigating widespread transmission [66].

Beyond forecasting, AI and big data enable real-time monitoring and global collaboration, which are essential for timely and coordinated responses to infectious threats. Traditional surveillance systems are often constrained by reporting lags and limited interoperability, whereas AI-powered platforms offer seamless data integration across multiple jurisdictions and sectors. For example, the Global Public Health Intelligence Network (GPHIN) and the Epidemic Intelligence from Open Sources (EIOS) initiative by the World Health Organization aggregate and analyze real-time data from a variety of sources, including news media, public health bulletins, and internet forums, to support early warning and response [52,67]. These platforms not only enhance timeliness but also promote transparency and international collaboration by providing a shared informational ecosystem.

Social media and news analytics also contribute significantly to real-time disease surveillance. Platforms such as Twitter, Facebook, and Google Trends furnish a vast stream of public data that, when processed using AI and NLP techniques, can reveal early signals of emerging health events. For instance, the analysis of social media posts mentioning symptoms or disease-related keywords has been used to predict influenza activity and monitor public sentiment during epidemics [68]. While not a replacement for clinical data, these unconventional sources offer complementary perspectives, especially in regions with weak health infrastructure or limited surveillance capacity [69].

The ability to integrate and process data from diverse sources is another major advantage of leveraging big data in disease surveillance. Unlike traditional systems that rely primarily on clinical and laboratory data, modern AI-enhanced systems can synthesize information from EHRs, genomic sequencing, environmental sensors, mobility data, and wearable technologies. The combination of genomic and epidemiological data, for instance, allows for the real-time tracking of pathogen evolution and transmission pathways, as seen in the use of genomic surveillance to identify SARS-CoV-2 variants of concern [70]. Geospatial data from satellite imagery and mobile phone tracking have also been used to monitor environmental conditions conducive to vector-borne diseases such as malaria and dengue, allowing for preemptive vector control interventions [71].

Moreover, mobile health applications and wearable devices are becoming increasingly valuable in gathering real-time physiological and behavioral data from individuals. These technologies can continuously monitor indicators such as heart rate, body temperature, and respiratory rate, which may signal early symptoms of infectious diseases [72]. The Apple Heart Study and similar digital health programs demonstrate how wearable data, when analyzed using AI, can contribute to public health surveillance by detecting population-level patterns that precede clinical diagnosis [73].

In the realm of diagnostics and clinical decision-making, AI has demonstrated its potential to improve accuracy and reduce human error. AI algorithms can analyze imaging, clinical, and laboratory data to aid in disease identification and triage. For example, during the COVID-19 pandemic, convolutional neural networks (CNNs) were used to differentiate COVID-19 from other forms of pneumonia on chest CT scans with high sensitivity and specificity [74]. In Ebola-endemic regions, AI-powered decision support tools have been employed to assist frontline workers in recognizing early signs of infection based on symptom clusters and epidemiological risk factors [75]. These applications not only enhance diagnostic confidence but also support clinicians in resource-limited settings where access to specialist expertise may be lacking.

AI’s role in improving diagnostic workflows also contributes to faster case identification and isolation, reducing transmission. By integrating data from wearable devices, symptom checkers, and telemedicine platforms, AI systems can support rapid diagnosis and remote monitoring, reducing the burden on healthcare facilities during outbreaks [76]. These innovations are especially critical in low- and middle-income countries (LMICs) where healthcare access is constrained and early detection can dramatically alter outbreak trajectories [77].

Another key opportunity lies in the cost-effectiveness and risk-strategic allocation of resources facilitated by AI and big data analytics. By identifying hotspots, predicting surges, and modeling intervention impacts, health authorities can better allocate limited resources such as vaccines, hospital beds, and personal protective equipment (PPE) [78]. Predictive models informed COVID-19 vaccination strategies by identifying priority populations and projecting the impact of various distribution scenarios [79]. During the Ebola outbreaks, AI-supported logistics systems helped optimize the delivery of medical supplies to affected areas based on predictive need assessments [80].

Moreover, cost savings accrue not only from more efficient use of supplies but also from averting large-scale outbreaks through timely interventions. Economic models suggest that AI-supported surveillance systems, while initially expensive to implement, offer substantial long-term savings by preventing disease spread, reducing hospitalization rates, and limiting economic disruption [81]. These benefits underscore the strategic importance of investing in digital infrastructure and AI capabilities as part of national and global health security frameworks.

Pitfalls: Challenges and risks of using big data and AI in disease surveillance

AI use across multiple sectors has given rise to numerous privacy issues. In the medical context, AI is used to interpret patient data for diagnostics, identify the most appropriate treatment, and monitor disease. However, the large-scale capture and processing of such data pose risks for privacy violations. Medical charts are, therefore, highly sensitive and require careful privacy protection to prevent unauthorized access and ensure patient confidentiality.

One of the main privacy threats is the inference attack. In this scenario, an attacker can infer sensitive information based on the model outputs and may be able to determine whether a specific dataset was used in training, leading to privacy infringements without direct data access [82]. Additionally, a significant challenge lies in data leakage during model training and inference. Such leaks, often due to inadequate security measures, can expose sensitive personal information to unauthorized users [83].

Another major concern is model re-identification, which undermines the privacy of anonymized datasets. By combining anonymized data with external datasets, attackers can re-identify individuals, rendering privacy protections ineffective [38]. These vulnerabilities emphasize the urgent need for the development of substantial privacy-preserving strategies in AI to safeguard user data and ensure compliance with ethical and legal standards.

Collecting and using personal health data also raises important ethical concerns, especially when it comes to autonomy and informed consent. When patients are unclear about how AI systems will use, share, or leverage their data, genuine informed consent becomes difficult to obtain, especially when consent is embedded in lengthy and complex digital terms of service [84]. This lack of understanding compromises patient autonomy and undermines the principle of voluntary participation in data sharing. For example, using health data to train algorithms or develop commercial products without explicit patient consent is unethical and potentially illegal [85]. Such practices can lead to stigmatization, identity theft, or denial of services such as insurance. The ethical obligation of “do no harm” reinforces the need for robust encryption and effective data access governance [86].

The development of effective AI models depends on access to large, high-quality health datasets. However, many developing countries are unable to generate or manage such datasets. Limited infrastructure, such as unreliable electricity, inadequate broadband internet, insufficient data storage, and the absence of digital health records, restricts the implementation of AI tools in low-resource settings [87]. In addition, the lack of standardized record-keeping systems and the underrepresentation of certain populations in global datasets contribute to data poverty. This hinders local innovation and exacerbates algorithmic bias on a global scale [88].

Accurate predictions from AI models are heavily dependent on the quality of input data. Yet, sources such as electronic health records, clinical notes, and patient-reported data may contain inaccuracies, inconsistencies, or outdated information. Errors introduced by human oversight, misclassification, coding mistakes, and transcription inaccuracies are especially problematic in the context of infectious disease surveillance, where precise case classification and rapid detection are vital [89,90].

Incomplete or poor-quality data can significantly impair AI performance in surveillance systems. As data move across different levels of care, these gaps become more pronounced. In low-resource settings, patient records may lack essential details such as laboratory confirmations, symptom onset, travel or contact history, and contact tracing information. Incomplete inputs can lead AI systems to underestimate case counts, overlook critical transmission clusters, or fail to detect emerging variants, such as novel COVID-19 mutations, which could pose serious risks during an epidemic or pandemic [91].

The issue of representativeness is another critical limitation. The surveillance model for most AI is trained using datasets from high-income countries, which may not reflect the demographics, disease patterns, or healthcare access realities of low- and middle-income countries (LMICs). Consequently, these models may fail to detect outbreaks or misestimate their severity in marginalized groups, including rural or displaced populations, thereby contributing to health inequities and delaying timely public health interventions [92,93].

Furthermore, health data drift over time, where training data are based on outdated clinical guidelines or past epidemiological patterns, can result in a mismatch between AI models and the evolving dynamics of disease. In the face of rapidly changing infectious disease landscapes, such as the progression of COVID-19 or emerging zoonotic threats, outdated training data can lead to inaccurate trend forecasts and inappropriate public health responses. Local variation in healthcare systems and reporting standards also hinders the transferability of AI models across different settings, limiting their global applicability [94].

These challenges, ranging from data privacy and informed consent to data quality and representativeness, undermine the efficiency, generalizability, and equity of AI-powered infectious disease surveillance. They highlight the urgent need for robust data governance, localized model development, and international collaboration in designing and implementing AI infrastructure for global health.

Case studies and applications

Case Study 1: AI and Big Data in COVID-19 Surveillance

In December 2019, SARS-CoV-2 emerged and became a catalyst for the application of AI in infectious disease monitoring. AI has been used for the prediction of emergence or re-emergence, tracking the spread of diseases, assisting in the diagnosis and prognostication of patients, and optimizing prevention and treatment protocols at a much faster rate than previously possible [34,95,96]. The global health crisis was declared over as a public health emergency of international concern (PHEIC) by the WHO in May 2023, with approximately 7.65 million cases and a 1% case fatality rate [95]. However, COVID-19 remains endemic in some locations.

Available data suggest that there were reports of six people presenting with symptoms suggestive of COVID-19 as early as December 8, 2019 [96]. BlueDot, an AI-powered system that monitors around 200 infectious diseases using machine learning algorithms, identified these and other cases as “unusual pneumonia” and issued notifications about a week prior to the official acknowledgment by epidemiologists and traditional disease surveillance systems that a novel coronavirus had emerged [34,96,97]. BlueDot employs a Feature Manipulation Engine (FME) to obtain and integrate health-related data from various sources, including government databases, health forums, notifications, electronic health records from centers for disease control, news reports, social media, and search engine queries [96,98]. It also predicted the potential spread of COVID-19 to other countries based on factors such as population density, perceived density of cases, historical data analysis, and available air travel data. Similarly, Metabiota analyzed the outbreak and accurately predicted that some of China’s neighboring countries were at high risk for virus spread, utilizing data from sources comparable to those used by BlueDot [96]. Older digital surveillance tools, such as ProMED-mail, also detected COVID-19 early [99].

In some regions, AI using machine learning and deep learning models, including neural networks and decision trees, contributed to the diagnosis of COVID-19 from unusual findings on chest X-rays and CT scans, demonstrating high sensitivity and specificity (90% and 100%, respectively) and thus surpassing the limitations of reverse transcription-polymerase chain reaction (RT-PCR) testing. Potential cases could also be identified through symptom tracking using wearable smart devices equipped with biosensors that detect coughing, fever, and respiratory distress and monitor vital signs, such as the Smart COVID-Shield. Additionally, AI-powered chatbots assessed symptoms and provided guidance for individuals who may have been infected. AI played a role in monitoring patients’ clinical progress and predicting severity and possible complications, such as the need for mechanical ventilation, ICU admission, the development of acute respiratory distress syndrome, sepsis, and the risk of rapid deterioration or death [34,97,99-104].

AI-powered contact tracing improved the early detection of potential cases. Machine learning algorithms analyzed available data from confirmed cases to identify potential contacts using digital applications that utilized Bluetooth Low Energy and GPS [97,98,105]. For example, Singapore’s TraceTogether application was installed on personal devices by the majority of COVID-19 cases (93.3%) in Singapore between August 2021 and February 2022. The use of this application reduced the time lag for reaching out to contacts of cases by about nine hours compared to manual contact tracing methods. However, only about half of the cases willingly uploaded their data to the application to facilitate digital contact tracing [106]. Johns Hopkins University developed an automated web-scraping tool to collect global data, organized it in a GitHub repository, and analyzed it using ArcGIS, a geographic information system (GIS) integrated with machine learning, to create a real-time dashboard mapping COVID-19 cases worldwide. This dashboard, updated twice daily, became the go-to resource for real-time case counts [34,59].

The rapid acquisition of predictions regarding case counts, disease spread patterns, early diagnosis, and mortality enabled the timely implementation of interventions such as nationwide lockdowns, restricted international travel, and other nonpharmaceutical interventions. These measures, including the use of masks, social distancing, handwashing, and mass testing, were employed to limit the spread of COVID-19 during the pandemic, well before and after vaccine development. Delays in such interventions could have resulted in much higher morbidity and mortality, unlike the SARS outbreak in 2002, which went undetected for four months and spread to 17 countries before being identified [96-98,107,108]. These developments underscore the critical role of AI in big data mining for analysis, prediction, and the suggestion of optimal solutions during infectious disease outbreaks.

AI and big data contributed significantly to the sequencing of the virus genome, identifying possible mutation patterns and potential antigen targets for vaccine development (through protein structure prediction and epitope mapping). AI facilitated the simulated screening of thousands of vaccine candidates, streamlined selection based on safety and efficacy, and optimized clinical trials, all within less than a year, compared to the usual 15-year timeframe for vaccine development [109-111]. This approach reduced costs, energy consumption, and the failure rate compared to traditional methods [109,111]. AI was also instrumental in identifying and screening potential drug candidates [97,112]. Furthermore, AI identified locations that should be prioritized for vaccine distribution using geospatial prioritization models based on outbreak density, predicted demand, and available guidelines. It also identified which high-risk individuals should receive the vaccine first, expediting the development of herd immunity and limiting waste while optimizing logistics such as storage temperature and transportation routes [113-116]. For example, Zipline used self-flying drones, relying on sophisticated AI-based route planning, to deliver vaccines to Ghana, with plans to expand to other African countries, including Nigeria, Rwanda, Kenya, and Côte d’Ivoire. These efforts notably improved delivery speed and access to vaccines in these countries [117,118]. AI also predicted potential adverse effects and provided real-time monitoring by integrating and analyzing medical reports and social media trends [109].

However, it can be argued that technological advancements were still insufficient to handle the pandemic adequately, as evidenced by the burden of morbidity and mortality and the time lag, though relatively short, between the emergence of the virus and its formal identification [34]. The reliability of big data, the accuracy of AI predictions, and the risk of over-reliance on AI remained a concern, especially since so little was known about the novel coronavirus at the outbreak’s onset. Questions remain as to how AI could have been properly trained to diagnose COVID-19 accurately or to identify high-risk locations and individuals for vaccine prioritization without bias, since AI relies on the quality and availability of data [34,98,109,114].

Additionally, concerns about data privacy are understandable in a world where vast amounts of data about individuals, institutions, and governments are readily available, and smart technologies such as AI can be used to obtain and analyze this information. The risks of misuse by bad actors are ever-present [109,118-120]. Big data may make sensitive information, such as home addresses or genetic details, accessible without consent. Many AI-powered applications and wearable devices must be connected to users’ mobile devices to be used for symptom tracking, vital sign monitoring, and location tracking, which could lead to the unintentional exposure of private information [105]. AI systems can monitor individuals’ movements, effectively enabling invasive surveillance. Some of these technologies are not cost-effective, making them inaccessible in low-resource settings, resulting in suboptimal surveillance, delayed diagnosis, and limited benefits from these innovations [97,105]. The rapid rate of vaccine development also contributed to vaccine hesitancy, driven by concerns about safety and the rise of conspiracy theories, despite AI’s ability to predict these reactions and suggest strategies to improve vaccine uptake [109].

Case Study 2: Predictive Analytics for Influenza Surveillance

Before the COVID-19 pandemic, big data analysis was already playing a crucial role in monitoring infectious diseases, and these capabilities have only improved with advances in AI. Influenza is responsible for up to one billion cases and 650,000 deaths annually. In 1997, the WHO launched FluNet, a web-based platform that collects influenza-related data weekly from national influenza centers and laboratories in over 130 countries. This system provides real-time global monitoring of influenza activity [121,122]. With the incorporation of machine learning models such as regression analysis, decision trees, support vector machines, and deep learning methods, FluNet now serves as an early warning system for potential influenza outbreaks, predicts spread and mutation patterns, and informs quicker public health responses [122].

Google Flu Trends was another predictive analytics system developed to forecast influenza activity based on search engine queries. Launched in 2008, it successfully predicted outbreaks up to two weeks earlier than traditional surveillance systems, using search terms such as “flu symptom,” “flu remedy,” and “fever remedy.” However, the system was shut down in 2015 after it greatly overestimated cases during the 2012-2013 outbreak and failed to improve its predictive performance [98,123]. This experience underscored that hybrid models are more effective for forecasting outbreaks, density, and spread since combining multiple AI models and analyzing data from different sources enhance accuracy and reliability [98,123,124]. This was especially evident during the COVID-19 era [96,98].

More advanced influenza surveillance tools include the CDC FluSight Network, which was launched in 2016. This system aggregates predictions from several academic teams into a single combined forecast for flu activity across the United States. It relies on machine learning models such as stacked regression and weighted averaging to improve accuracy [125]. The system utilizes a variety of data sources, including clinical surveillance reports, digital data from search engine trends, and electronic health records, as well as environmental factors such as humidity and temperature patterns [125].

Another example is DeepFlu, a hybrid model introduced in 2019. DeepFlu combines classical statistical methods (such as ARIMA) with machine learning models such as LSTM neural networks [124]. Similarly, Inferno is a Bayesian machine learning model created to predict flu activity and provide estimates with confidence intervals [126]. Research continues to propose more improved models for influenza surveillance [127].

Case Study 3: The Role of Big Data in Zika Virus Monitoring

Big data analysis has also been essential in the surveillance of the Zika virus and in developing effective strategies for its prevention and control. Zika was declared a PHEIC in 2016 due to its rapid spread during the outbreak that began in 2015 and possibly even earlier in Brazil, the Americas, Asia, and West Africa [128,129]. The virus was particularly concerning due to its association with long-term complications such as anencephaly in fetuses of pregnant women who became infected, as well as other neurological sequelae [128].

Initial efforts at surveillance and containment in 2015 were inadequate, which allowed Zika to spread to about 25 countries before it was declared a PHEIC and before the need for improved surveillance methods was recognized [128,129]. During the outbreak, data were analyzed using computational, mathematical, and geospatial models to understand transmission patterns and the factors that favored vector breeding. Geographic information systems (GIS), used by tools such as ProMED-mail and HealthMap, were employed to map the habitats of mosquito vectors and predict high-risk locations for Zika virus cases based on climate conditions, proximity to mosquito habitats, population density, and travel patterns. These methods supported more targeted interventions [128].

Research has shown that mobile applications were created to track Zika cases [130,131]. In addition, social media trends could have been combined with traditional surveillance for the faster prediction of outbreaks, and social media played a significant role in public health campaigns [129,132]. Despite these advances, the accurate surveillance and prediction of Zika virus outbreaks remain challenging. The ongoing evolution of the virus, changes in its mosquito vector, climate variability, a high number of asymptomatic cases, and persistent issues with delayed and poor-quality data continue to hinder efforts at accurate forecasting [130].

Recommendations for future research and implementation

The future application of artificial intelligence in infectious disease surveillance depends not only on technological advancements but also on the thoughtful integration of systems, policies, and people. As interest in AI continues to grow, future research and implementation must focus on strengthening foundational areas that enable its meaningful and ethical use in global public health.

One of the most critical priorities is improving data integration and sharing. The strength of AI tools lies in their ability to analyze vast amounts of data, yet these tools are only as effective as the quality and completeness of the information they receive. Currently, health data are often fragmented, inconsistent, and siloed across different regions and institutions. Establishing centralized and collaborative data platforms can help address this challenge. Such platforms should be designed to allow the timely and secure exchange of public health information across countries. They must also be sensitive to local contexts, accommodating regional variations in disease patterns, health infrastructure, and reporting practices. International public health agencies, in collaboration with digital health experts, should lead the creation of standardized data protocols that guide collection and sharing, with a strong emphasis on ensuring that data are representative and locally relevant [133].

Achieving this level of data integration requires substantial investment in technological infrastructure, particularly in low-resource settings. Many countries still lack the digital backbone needed to support advanced surveillance systems. Investments should focus not only on internet connectivity and data storage but also on building institutional capacity and training skilled personnel. Ensuring that every country can collect, store, and analyze its own data will strengthen both national preparedness and the global response to emerging health threats [98]. These investments must be sustainable and equitable, avoiding the risk of widening the digital divide in public health.

Ethical and legal considerations must also be addressed with greater urgency. As AI becomes more involved in health decision-making, questions surrounding data privacy, informed consent, and algorithmic accountability become increasingly complex. There is a pressing need for clear, enforceable legal frameworks that define the acceptable use of AI in public health contexts. These frameworks should be as robust and universally respected as established human rights laws. Existing policies can be revised and expanded, but where gaps exist, new legislation must be developed through inclusive and multidisciplinary processes. Such frameworks will help safeguard individual autonomy while offering practical guidance for researchers, policymakers, and healthcare providers [99].

In addition to policy and infrastructure, attention must be given to how AI is integrated into clinical and public health practice. While AI can offer valuable support, it should never replace the critical thinking and contextual judgment of human professionals. Over-reliance on algorithms may lead to missed nuances, especially in complex or ambiguous situations. To ensure responsible use, medical education should include structured training on the fundamentals of AI and digital health. This training should be incorporated into formal curricula rather than offered as optional seminars. Equipping healthcare workers with the knowledge to understand and evaluate AI tools will allow them to use these technologies more safely and confidently. The goal should be a collaborative model in which AI supports clinical decision-making while leaving final responsibility with trained professionals [134].

## Conclusions

Big data and artificial intelligence have introduced unprecedented opportunities to strengthen infectious disease surveillance. These technologies enhance the ability to detect outbreaks early, integrate complex data sources, and guide timely public health responses. From forecasting influenza trends to managing COVID-19 spread, AI has demonstrated tangible value in supporting global health initiatives. Nevertheless, the implementation of these tools is accompanied by ethical, technical, and operational challenges. Safeguarding data privacy, addressing inequities in digital access, and maintaining transparency in algorithmic decision-making are crucial considerations. Over-reliance on technology without adequate human oversight risks undermining the accuracy and accountability of surveillance efforts.

Moving forward, public health systems must invest in equitable digital infrastructure, promote responsible data governance, and prioritize training that bridges the gap between human expertise and AI capabilities. By adopting a thoughtful and collaborative approach, the integration of big data and AI can be harnessed to build more resilient, responsive, and inclusive disease surveillance systems.
